# Increased risk of brain metastases in women with breast cancer and p16 expression in metastatic lymph-nodes

**DOI:** 10.18632/oncotarget.16953

**Published:** 2017-04-08

**Authors:** Elise Furet, Morad El Bouchtaoui, Jean-Paul Feugeas, Catherine Miquel, Christophe Leboeuf, Clémentine Beytout, Philippe Bertheau, Emilie Le Rhun, Jacques Bonneterre, Anne Janin, Guilhem Bousquet

**Affiliations:** ^1^ Université Paris Diderot, Inserm, Paris, France; ^2^ INSERM, U1165-Paris, France; ^3^ INSERM, U1137-Paris, France; ^4^ Université de Franche-Comté, Département de Biologie, Besançon, France; ^5^ Hôpital Saint-Louis, APHP, Service de Pathologie, Paris, France; ^6^ Centre Oscar Lambret, Département de Sénologie, Lille, France; ^7^ CHRU de Lille, Neuro-oncologie, Département de neurochirurgie, Lille, France; ^8^ INSERM, Villeneuve d’Ascq, France; ^9^ Université Lille 2, Département de Sénologie, Lille, France; ^10^ Université Paris 13, Service d’Oncologie, Villetaneuse, France; ^11^ Hôpital Avicenne, APHP, Service d’Oncologie Médicale, Bobigny, France

**Keywords:** brain metastases, breast cancer, p16

## Abstract

**Purpose:**

Metastatic breast cancer is a leading cause of mortality in women, partly on account of brain metastases. However, the mechanisms by which cancer cells cross the blood-brain barrier remain undeciphered. Most molecular studies predicting metastatic risk have been performed on primary breast cancer samples. Here we studied metastatic lymph-nodes from patients with breast cancers to identify markers associated with the occurrence of brain metastases.

**Results:**

Transcriptomic analyses identified *CDKN2A/p16* as a gene potentially associated with brain metastases.

**Materials and Methods:**

Fifty-two patients with HER2-overexpressing or triple-negative breast carcinoma with lymph nodes and distant metastases were included in this study. Transcriptomic analyses were performed on laser-microdissected tumor cells from 28 metastatic lymph-nodes. Supervised analyses compared the transcriptomic profiles of women who developed brain metastases and those who did not. As a validation series, we studied metastatic lymph-nodes from 24 other patients.

Immunohistochemistry investigations showed that p16 mean scores were significantly higher in patients with brain metastases than in patients without (7.4 vs. 1.7 respectively, *p* < 0.01). This result was confirmed on the validation series. Multivariate analyses showed that the p16 score was the only variable positively associated with the risk of brain metastases (*p* = 0.01).

With the same threshold of 5 for p16 scores using a Cox model, overall survival was shorter in women with a p16 score over 5 in both series.

**Conclusions:**

The risk of brain metastases in women with HER2-overexpressing or triple-negative breast cancer could be better assessed by studying p16 protein expression on surgically removed axillary lymph-nodes.

## INTRODUCTION

Metastatic breast cancer is a leading cause of mortality in women, with a survival from 8.8 to 34.4 months [1], depending on metastasis distribution and histological sub-type. Brain metastases are associated with the poorest survival (median under 15 months) [2]. They occur in the progression of 15 to 40% of metastatic breast cancers. A high incidence of brain metastases is associated with HER2 and triple-negative subtypes [3, 4].

There is inadequate transfer of chemotherapeutic agents through the blood-brain barrier, while cancer cells are able to cross it and to invade the brain. Surgical access to brain metastases is also difficult, and very few studies have been performed on metastatic samples. Most molecular studies predicting metastatic risk (including brain metastatic risk) have been performed on primary breast cancer samples [5–9], and characterize the molecular signature of majority clones within the primary tumor. But primary breast cancers are heterogeneous [10, 11], and metastases derive from selected aggressive clones that have acquired resistance to first line treatment [12]. These clones, which may be a minority in the primary tumor [13], are precisely those on which genomic analyses need to be performed to guide targeted therapies.

This study was performed on laser-microdissected tumor cells from metastatic lymph-nodes from patients with metastatic triple-negative or HER2 breast cancer. Clinical follow-up enabled us to compare molecular results in women who developed brain metastases and in those who did not. Our aim was to identify biomarkers associated with the occurrence of brain metastases over a median follow-up of 2 years.

## RESULTS

### Characteristics of the 28 patients with transcriptomic analyses of laser-microdissected metastatic lymph-nodes

Among the 28 patients with metastatic HER2 or triple-negative breast cancer (Supplementary Figure 1 for patient selection), 15 had brain metastases, and 13 did not (Table 1). The 28 metastatic lymph-node samples were all imagery-guided pre-treatment biopsies of one axillary or one supra-clavicular lymph-node per patient, obtained at the time of localized disease or metastatic relapse (Supplementary Table 1). All biopsies were performed with a 16-gauge needle providing samples with a mean length of 13.11 mm (± 1.52 mm), mean width 1.11 mm (± 0.10 mm), and mean surface area 14.65 mm^2^ (± 1.78 mm^2^). Each metastatic lymph-node was laser-microdissected to specifically select a minimum number of 1000 tumor cells, with a mean surface area of 59.9mm^2^ (35.0 to 79.9 mm^2^). After RNA extraction, all samples were of good quality, enabling transcriptomic analyses, since the mean RNA integrity number was 8.6 (range 7–10).

**Table 1:** Characteristics of the 28 patients with transcriptomic analyses of metastatic lymph nodes

	With brain metastases *n* = 13	Without brain metastases *n* = 15	*p*-value
Median age at diagnosis (range)	53 years (38–70)	56 years (26–65)	ns
Median survival from diagnosis of metastatic disease (range)	22.7 months(0.5 – 74.1)	34.4 months(14.2–80.3)	< 0.01
Histological sub-type: *n* (%)			
HER2-overexpressed	5 (38.5)	7 (46.5)	ns
Triple negative	8 (61.5)	8 (53.5)	

From diagnosis of metastatic disease, median survival was significantly shorter for the 15 women with brain metastases (22.7 months *vs*. 34.4 months, *p* < 0.01).

For the sub-group with triple negative breast cancer, median survival was not significantly different for women with and without brain metastases (15.8 *vs*. 17.9 months). For the sub-group with HER2-overexpressing breast cancer, median survival was significantly shorter for women with brain metastases (42.0 vs. 72.7 months, *p* < 0.01).

From the moment metastases were diagnosed in the brain, the median survival was very short, only 4.2 months, ranging from 0.3 to 23.4 months.

### *CDKN2A/p16* gene expression in metastatic lymph-nodes of women with brain metastases

Transcriptomic analyses were performed on laser-microdissected tumor cells from metastatic lymph-nodes of the 28 women with HER2 or triple-negative breast cancer. Multivariate analysis was carried out to compare data from women with brain metastases and those without. Table 2 shows the genes with the greatest fold changes between the two groups, and corresponding p-values adjusted for other metastatic localizations. *CDKN2A*, also called *p16^ink4a^*, evidenced a fold change of 2, the highest value (at the top of Table 2).

**Table 2:** Genes differentially expressed in metastatic lymph nodes of women with versus without brain metastasis

Gene	Without brain metastasis (mean)	With brain metastasis (mean)	Fold Change	*p*-value	FDR
**CDKN2A**	**10,1**	**12,1**	**2**	**4,5E-03**	**3,5E-02**
CRYBA2	7,1	8,9	1,8	4,9E-02	1,0E-01
DMKN	9,7	11,3	1,6	9,6E-03	6,2E-02
CHST8	7,9	9,4	1,5	2,5E-02	9,6E-02
SEZ6L2	10,6	12,1	1,5	1,5E-03	2,9E-02
LOC283454	6,7	8,1	1,4	3,0E-02	9,8E-02
ACTL8	7,4	8,8	1,4	4,9E-02	1,0E-01
HRK	7,4	8,7	1,4	4,4E-02	1,0E-01
DMKN	7,9	9,2	1,3	1,3E-02	7,1E-02
ASPHD1	8,4	9,6	1,2	1,7E-02	7,5E-02
PAQR6	10,1	11,3	1,2	4,2E-03	3,5E-02
SNORA76	8,5	9,8	1,2	4,7E-02	1,0E-01
NKD2	9,2	10,3	1,2	1,4E-02	7,1E-02
MMP11	9,5	10,7	1,2	2,8E-02	9,8E-02
LOC100289092	8,2	9,3	1,1	3,0E-03	3,5E-02
IGDCC3	6	7,1	1,1	4,8E-02	1,0E-01
LOC100130547	6,9	8	1,1	8,5E-04	2,9E-02
CACNA2D2	6,5	7,5	1,1	4,0E-02	1,0E-01
TBC1D9	8,8	9,9	1,1	4,1E-02	1,0E-01

The table indicates genes with a fold change more than one. Mean logarithmic values in the two groups are shown. *p*-values are computed from a multivariate linear regression analysis (adjusted with subtype of cancer and other metastatic locations). Association was considered as significant if the FDR (false discovery rate) was less than 0.05.

Using RT-qPCR on all 28 metastatic lymph-nodes, we confirmed that *CDKN2A* mRNAs was significantly more expressed in women who developed brain metastases than in women who did not (RQ = 4.3 for *CDKN2A*, *p* < 0.01, Supplementary Figure 2).

We checked whether the association between *CDKN2A* expression in metastatic lymph-nodes and the risk of brain metastases could also be found in primary breast cancers. Available data from previously published studies were pooled and analyzed (Supplementary Table 2, and Supplementary Excel Files 1 and 2), but the data did not show any association between *CDKN2A* expression level in primary breast cancers and the risk of brain metastases (*p* = 0.16).

### p16 protein expression in metastatic lymph-nodes is also associated with the risk of brain metastases

**Figure 1:**
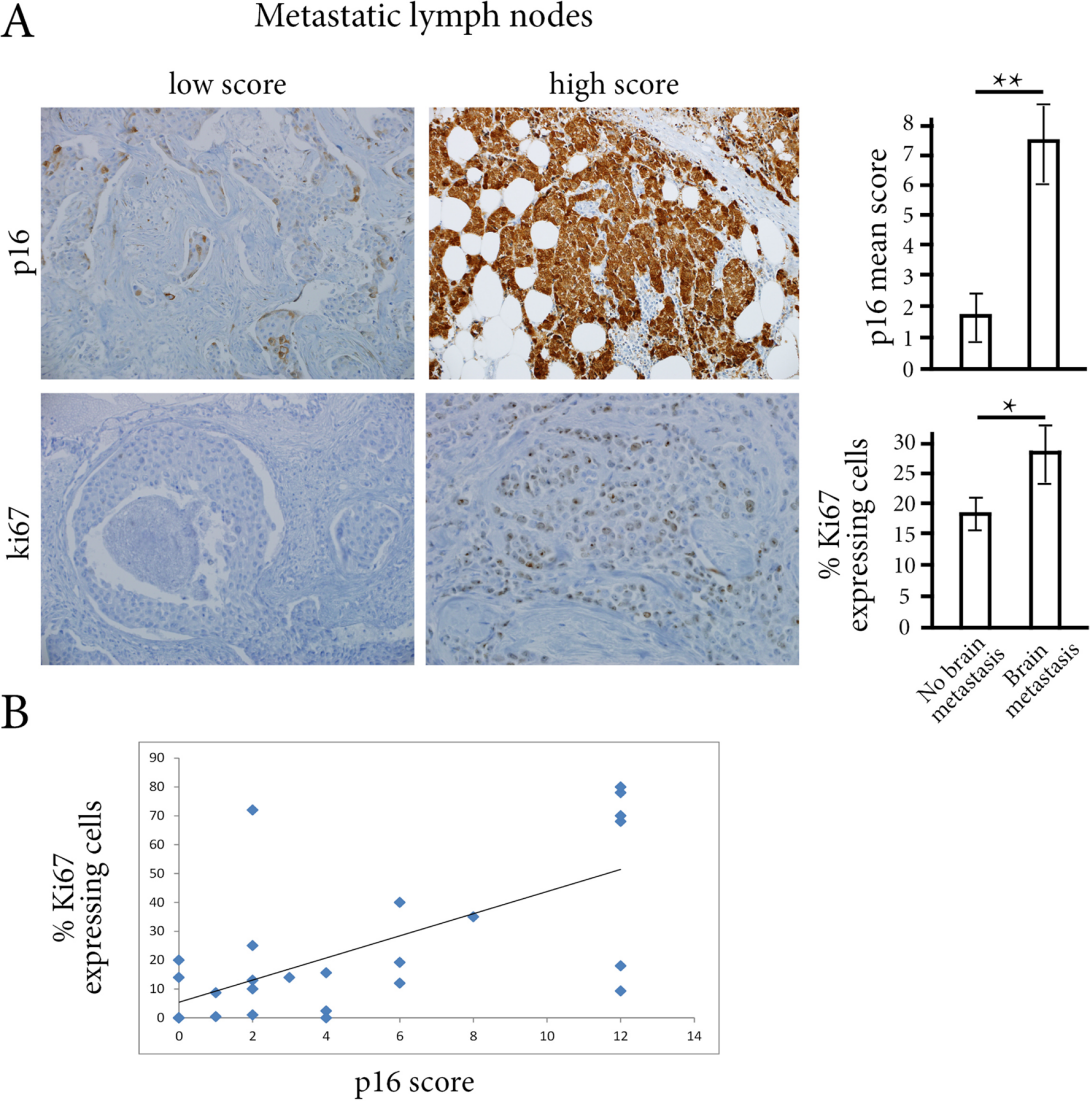
(**A**) Using immunostainings on metastatic lymph-nodes, p16 score and the percentage of Ki67-expressing cells were significantly higher in women with brain metastases than in women without brain metastases. **p < 0.01, *p < 0.05 (**B**) There is a correlation between p16 score and the percentage of Ki67-expressing tumor cells (R Pearson coefficient of 0.58, *p* < 0.01).

Using immunohistochemistry, we assessed p16 expression on the 28 metastatic lymph-nodes. We also wondered whether a high p16 score might be associated with cell cycle progression and thus with increased risk of brain metastasis. We therefore decided to assess the Ki67 proliferation marker on the same metastatic samples. We found higher p16 scores and larger numbers of Ki67-expressing cells for women with brain metastases than for women without brain metastases (7.4 *vs*. 1.7 respectively for p16 score, *p* < 0.01; 29% *vs*. 18%, respectively for Ki67, *p* < 0.05) (Figure 1A). There was also a correlation between p16 and Ki67 expression in tumor cells (Pearson correlation coefficient R = 0.58, *p* < 0.01) (Figure 1B).

**Figure 2:**
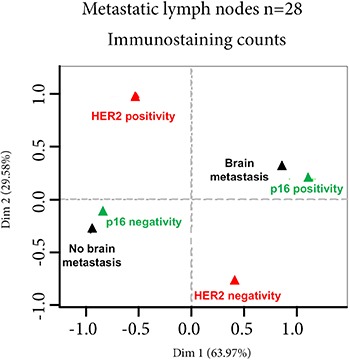
multivariate analyses Multivariate analysis of p16 score, percentage of Ki67-expressing cells, HER2 status, estrogen receptor (ER) and progesterone receptor (PR) status of metastatic lymph-nodes, and metastatic distribution other than in lymph-node shows that only the p16 score is significantly associated with the risk of brain metastases. *p < 0.01.

We then studied p16 scores, percentages of Ki67-expressing cells, HER2 status, and estrogen receptor and progesterone receptor status on metastatic lymph-nodes for their correlation with metastasis occurrence elsewhere than in lymph-nodes (brain, lung, bone or liver metastases). Univariate analysis showed that the p16 score and the percentage of Ki67-expressing cells were significantly associated with the risk of brain metastases (*p* = 10e-4 and 2.6×10e-2 respectively, Table 3). Variable selection by multivariate forward regression showed that the p16 score was the only variable positively associated with the risk of brain metastases (*p* = 0.01). A multiple correspondence analysis clearly showed this association between p16 positivity and brain metastasis (Figure 2). Using a threshold of 5, sensitivity and specificity of the p16 score for the risk of brain metastases were respectively 78.6% and 100% (Supplementary Figure 3).

**Table 3:** Univariate analyses on metastatic lymph nodes for the correlation with distribution of metastases (brain, lung, bone or liver)

	Brain metastases			Lung metastases			Bone metastases			Liver metastases		
	Yes	No	*p*-value	Yes	No	*p*-value	Yes	No	*p*-value	Yes	No	*p*-value
HER2 overexpression (% cases)	54	36	ns	31	57	ns	40	47	ns	56	39	ns
ER positivity (% cases)	8	21	ns	23	7	ns	20	12	ns	0	22	ns
PR positivity (% cases)	8	7	ns	8	7	ns	20	0	ns	0	11	ns
**%Ki67-expressing tumor cells**	**28**	**18**	**< 0.05**	24	22	ns	20	28	ns	21	28	ns
**p16 score in tumor cells**	**7**	**1.7**	**< 0.001**	6	3	ns	4	6	ns	5	5	ns

### Validation series for p16 expression and survival

Twenty-four metastatic lymph-nodes from women with metastatic HER2 or triple-negative breast cancer from Centre-Oscar-Lambret (Lille, France) were studied as a validation series. Sixteen women had clinically-diagnosed brain metastases, and eight did not (Supplementary Table 3).

When we assessed p16 protein expression on metastatic lymph-node sections, we found a mean score of 8.3 (± 3.3) for women who developed brain metastases, significantly higher than the mean score of 2.8 (± 1.9) for women who did not develop brain metastases (*p* < 0.01).

Using the threshold of 5 for the p16 score, sensitivity and specificity for the risk of brain metastases were respectively 81% and 88%.

**Figure 3:**
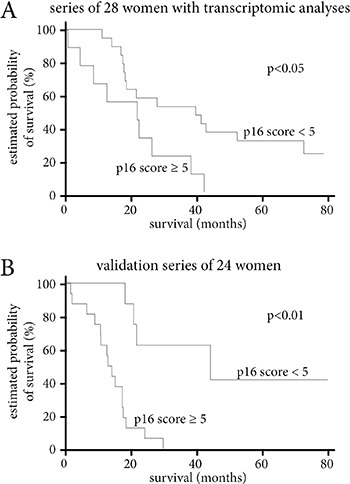
Survival analyses according to p16 score (**A**) Survival according to p16 score level in the series of 28 women with transcriptomic analyses. (**B**) Survival according to p16 score level in the validation series of 24 women. Survival according to p16 score level of the 52 women in the two series combined. In all three analyses, a p16 score over 5 is associated with a significantly shorter median survival.

When we used the same threshold of 5 for p16 score with a Cox model to analyze the two patient series separately, there was shorter overall survival in women with a p16 score over 5 in both series. For the initial series of 28 patients, median survival was 42.1 months in case of p16 score of 5 or under, and 21 months in case of p16 score over 5 (HR = 2.9, IC95% [1.2–7.1], *p* < 0.05) (Figure 3A). For the validation series, median overall survival was 43.2 *versus* 16.6 months respectively (HR=8.1, IC95% [2.3–29], *p* < 0.01) (Figure 3B).

Overall, our results showed an increased risk of brain metastases and shorter survival in women with breast cancer and p16 positivity in metastatic lymph-nodes.

## DISCUSSION

Here, we identified p16 as a marker significantly associated with the risk of brain metastases in women with HER2-overexpressing or triple-negative metastatic breast cancers.

*CDKN2A* is a tumor-suppressor gene encoding for the p16^ink4a^ protein. p16^ink4a^ inhibits cyclin-dependent kinases CDK4 and 6 [14], and prevents phosphorylation of *retinoblastoma* tumor suppressor (*RB*). *RB* represses the activity of the E2F family of transcription factors and blocks cell cycle progression (Supplementary Figure 4). When *CDKN2A* is lost and *RB* is normally functional, RB phosphorylation enables cell cycle progression. p16 is lost in about 30% of breast cancers, mainly as a result of genetic or epigenetic inactivation of *CDKN2A* [15, 16], but its prognostic value in these cases has not been fully demonstrated.

Recent studies on the *RB* pathway have shown that RB protein can be lost by genetic or epigenetic inactivation of the *retinoblastoma* gene, which in turn paradoxically induces high levels of p16^ink4a^ [17, 18]. A high expression level of p16^ink4a^ protein is a hallmark of HPV-related cervical and head-and neck carcinomas, secondary to RB inactivation by the HPV E7 protein [19].

*CDKN2A* deletions have frequently been found in melanoma [20] and lung cancer [21] brain metastases, suggesting a potential role of *RB* pathway inactivation and metastatic diffusion to the brain. In breast cancer, the relationship between p16 expression and the risk of brain metastasis has not been reported so far.

A higher lymph-node metastatic risk has been associated with p16 protein expression in women with triple-negative localized breast cancer [22]. In 71 localized breast cancers, high p16 protein expression was linked to triple-negative and RB-negative phenotype, and associated with an increased proliferation index [23]. In our metastatic lymph-nodes in triple-negative and HER2 breast cancers, we also found a correlation between p16 scores and the proliferation index. However, in multivariate analysis, for the two histological subtypes, only the p16 score was significantly associated with the risk of brain metastases.

When we performed a pooled analysis on published data from 787 primary breast cancers in women who developed brain metastases and others who did not, there was no association between *CDKN2A* expression and the occurrence of brain metastases. These pooled data were obtained from primary tumors, and could thus underestimate minority p16-positive clones with metastatic potential. The concept of intra-tumor heterogeneity, identified in renal cell carcinomas [24], is now challenging oncologists managing breast cancers [10], particularly to decipher the mechanisms of metastatic disease. Using laser-microdissection and molecular analyses of tumor cells in primary renal cell carcinoma and metastatic samples, we were able to track back a minority clone with a *TP53* mutation in the primary tumor, secondarily expanded in lung metastases [13].

In the present study, we focused on breast cancer metastatic lymph-nodes and performed transcriptomic analyses on tumor cells selected by laser-microdissection. The molecular analyses were thus specifically performed on metastatic clones, strengthening the validity of our genomic results. A main limitation could be the sample size of our two series. However, very few genomic studies have been performed on metastatic samples of breast cancer; they have usually been smaller in size and performed on tumor samples that were not laser-microdissected [25–28]. In a larger series of 80 patients [29], the samples were fine-needle aspirates and the histological sub-type was unknown, more than 60% matched-primary tumors being estrogen-receptor positive. In our study, only triple negative and HER2-overexpressing breast cancers were considered.

The main result obtained in our study was the relationship between the p16 score in metastatic lymph-nodes and brain metastases, independently from the tumor sub-type (HER2- or triple negative).

This result may have a strong clinical application, since a systematic assessment of p16 protein expression on surgically-removed axillary lymph-nodes is simple – and not costly – to implement, to better assess the risk of brain metastases in women with HER2-overexpressing or triple-negative breast cancer.

In case of p16 protein expression in metastatic lymph-nodes, the far more costly screening for brain metastases by magnetic resonance imaging could be considered, since early detection of occult brain metastases significantly decreases the risk of death linked to brain metastases (from 48% to 16%) in women with metastatic breast cancer [30].

## MATERIALS AND METHODS

### Patient data

Fifty-two patients from two different hospitals with available tumor samples and follow-up data were included in this study (28 from Saint-Louis Hospital, Paris corresponding to the experimental set, and 24 from Centre-Oscar-Lambret, Lille corresponding to the validation series).

In compliance with French Bioethics law (2004–800; June 8, 2004), all patients had been informed of the research use of the part of their samples remaining after diagnosis had been established, and none opposed it. Informed consent was obtained from each patient. The Clinical Research Board Ethics Committee approved this study (CPP-Ile-de-France#13218).

All patients, diagnosed between 2003 and 2013, had metastatic HER2 or triple-negative breast carcinoma either at initial diagnosis or during follow-up of the disease and classified M1 according to TNM classification [14].

Based on clinical and imaging data, and a median follow-up of 24 months from diagnosis of metastatic disease, we separated women with and without brain metastases. Among patients without brain metastases, those with a survival or a follow-up of less than 14 months were excluded. We chose this threshold because the median time between metastatic disease and occurrence of brain metastases ranges from 6 to 9 months for women with metastatic breast cancer [15, 16], and probably even more for HER2 subtype [17].

### Laser-microdissection of tumor cells from metastatic lymph-nodes and transcriptomic data processing

After consultation of the Pathology Department register (Saint-Louis Hospital), we identified 359 patients with frozen metastatic samples collected between 2003 and 2013, 143 being lymph node metastases from breast cancers. Among women with HER2- or triple negative sub-type and distant metastases during follow-up, we identified 32 women with available frozen tumor samples (Supplementary Figure 1).

Cryo-cut sections of these 32 frozen metastatic lymph-nodes from 32 patients in Saint-Louis- Hospital were laser-microdissected to select tumor cells in the samples. Using a PALMMicrobeam/Zeiss-system, a minimum of 1000 tumor cells were laser-microdissected on 7 μm-thick tissue sections for a minimum surface area of 35.0 mm^2^ for each metastatic lymph-node.

Total RNA was extracted from laser-microdissected tumor cells using RNeasy-Mini-Kit (Qiagen, France), quantified on NanoDrop and qualified on Bio-Rad-ExperionTM Automated- Electrophoresis-Station (BioRad, France). Four out of the 32 cases had RNA of insufficient quality.

The remaining 28 cases had a mean RNA integrity number of 8.6 (range 7–10), and corresponded to the 28 women in the experimental series.

Transcriptomic analyses were performed using MiltenyiBiotec-Microarray service. A linear T7- based amplification step was performed on 0.5 μg of all RNA samples. To produce Cy3-labeled cRNA, the RNA samples were amplified and labeled using Agilent-Quick-labeling kit. Yields of cRNA and dye-incorporation rates were measured with ND-1000-Spectrophotometer (NanoDrop, LabTech, France). Hybridization was performed according to Agilent 60-mer-oligomicroarray protocol: 1.65 μg Cy3-labeled cRNA were hybridized (overnight/65°C) on Agilent-Whole-Human-Genome-Oligo-Microarrays 8 × 60K V2, and fluorescence signals detected using Agilent-Microarray-Scanner. Agilent-FE-Software determined feature intensities. Quantile normalization was performed using the limma package on R-software version 3.2.1 (Foundation for Statistical Computing, Vienna, Austria), based on log2 single-intensity expression data.

### RT-qPCR and validation of *CDKN2A* mRNA expression in metastatic lymph-nodes

On following sections of the same laser-microdissected metastatic lymph-nodes, we used RTqPCR to validate the transcriptomic results for *CDKN2A* [Hs00923894_m1] and *APC2* [Hs_00183420m1].

Total RNA was reverse-transcribed (cDNA) before qPCR amplification using random primers with SuperScript-TM-II-Reverse Transcriptase (Invitrogen, France). The qPCR reactions were performed using fluorescent probes on a CFX96 Real-Time-System (Bio-Rad) according to MIQE guidelines [18]. A blank sample with no cDNA was included, and the experiments were performed in triplicate for each gene, each sample being duplicated on the PCR plate. The reference gene *TBP* [Hs99999910_m1] was used to normalize gene expression results. The results were expressed as 2-ΔΔCq (relative quantification).

### *In situ* p16 expression on metastatic lymph-nodes

Using immunochemistry, p16 expression was assessed on the metastatic lymph-nodes: i) in the series of 28 lymph-nodes already processed for transcriptomic analyses; ii) in a validation series of 24 axillary lymph-nodes from Centre-Oscar-Lambret (Lille, France).

An indirect immunoperoxidase method using anti-p16ink4a (E6H4,Roche Diagnostics, Meylan, France) as the primary antibody was performed on 5 μm-thick tissue sections of each metastatic lymph-node. The secondary antibody was a rabbit-monoclonal anti-mouse IgG1 (M1gG51-4, Abcam, UK) coupled with antirabbit OmniMap detection kit (Roche-Diagnostics).

Systematic controls were absence of primary antibody and use of an irrelevant primary antibody of the same isotype.

For each tissue section, cells expressing p16 were counted by two different pathologists (GB, AJ) on five different fields at ×400 magnification, using a ProvisAX70 microscope (Olympus, Tokyo) with wide-field eyepiece number 26.5, providing a field size of 0.344mm2 at this magnification.

A cytoplasmic and nuclear distribution of p16 was considered positive. For each field, a minimum of 100 tumor cells were analyzed. The percentage of p16-expressing cells was the number of positive cells in these 100 tumor cells.

Each sample was given a score [19] by multiplying the stain intensity grade (0 = no staining, 1 = low intensity, 2 = medium intensity, 3 = strong intensity) by the numerical code for the percentage of positive cells (0 = 0%, 1 = under 10%, 2 = 10–50%, 3 = 51–80%, 4 = over 81%). The maximum score was 12 when more than 81% of the cells expressed p16 with a strong intensity signal. Results were expressed as mean ± standard error of the mean (SEM).

### Statistical analyses

Univariate and multivariate analyses were carried out with R-software version 3.2.1 (Foundation-for-Statistical-Computing, Vienna, Austria).

For analyses performed on our transcriptomic data, the genes associated with brain metastases were identified by multivariate linear regression using the “glm” R-function. With this model, brain metastasis was the dependent variable and association with gene expression could be adjusted on other metastatic localizations, on biopsy site, and on ESR1 and ERBB2 status. This made it possible to look for genes associated with brain metastasis independently from these factors.

*p*-values were corrected for multiple comparisons using Benjamini-Hochberg correction which provided false discovery rates (FDRs, calculated with the “p.adjust” R-function).

We performed the same analyses on transcriptomic data downloaded from public databanks in which annotations for brain metastases were available. All these transcriptomic data were obtained for primary breast cancers, and downloaded from Gene-Expression-Omnibus website (GSE2603 [20], GSE2034 [6], GSE7390 [21], GSE12276 [8]). 583 transcriptomes obtained with HG-U133A GeneChip arrays (Affymetrix) and 204 transcriptomes produced with HG-U133A plus 2.0 GeneChip arrays (Affymetrix) were separately analyzed after quantile normalization.

For graphic representations and correspondence analyses, transcript levels of genes such as *ESR1*, *ERBB2* or *CDKN2A* were discretized into binary variables (positive or negative) using the Fisher-Jenks algorithm (classInt R-package).

Correspondence analyses between qualitative variables were performed with the FactoMine Rpackage.

To specifically focus on associations between two variables, we used the chi-square test (for qualitative variables) or the *t*-test (for quantitative variables).

For the correlation study between p16 immunostaining scores and Ki67 expression levels in metastatic lymph-nodes, the Pearson correlation coefficient was calculated.

Association between p16 immunostaining scores and overall survival was performed using a Cox model with the “survival” R-package. Two data sets were studied separately with univariate and multivariate tests, one series corresponding to the transcriptomic study including 28 lymph-nodes and the other was the validation series including 24 metastatic lymph-nodes. Overall survival was calculated from the date of metastatic disease.

## SUPPLEMENTARY MATERIALS FIGURES AND TABLES







## Abbreviations

SEM: standard error of the mean
FDR: false discovery rate
RB: retinoblastoma.
