# Supplementary material for: Increased risk of brain metastases in women with breast cancer and p16 expression in metastatic lymph-nodes
Source: Oncotarget. 2017 Apr 8;8(23):37332–41. doi: 10.18632/oncotarget.16953 (PMC5514912; doi:10.18632/oncotarget.16953)
Supplement: Supplementary file 1 [file oncotarget-08-37332-s001.pdf]

Increased risk of brain metastases in women with breast cancer and p16 expression in metastatic lymph-nodes

Supplementary Materials

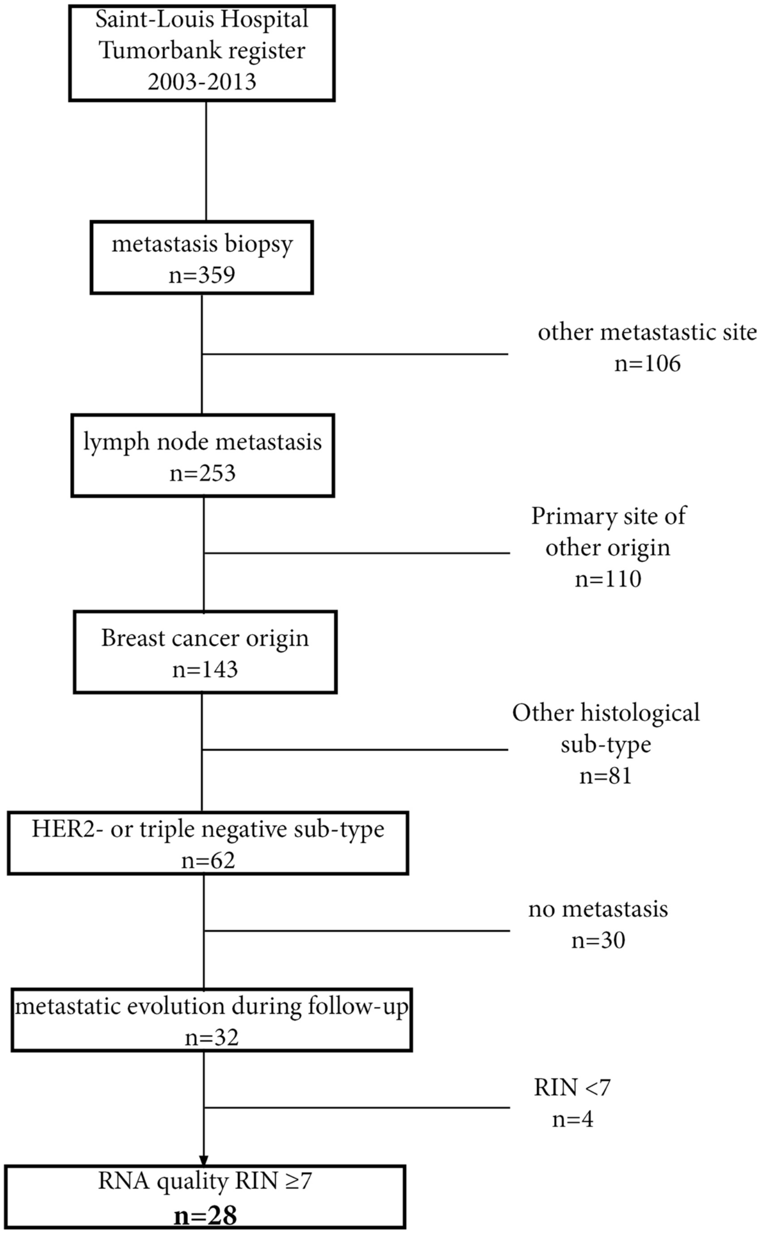

Supplementary Figure 1: Diagram for selection criteria of the 28 patients of the experimental series.

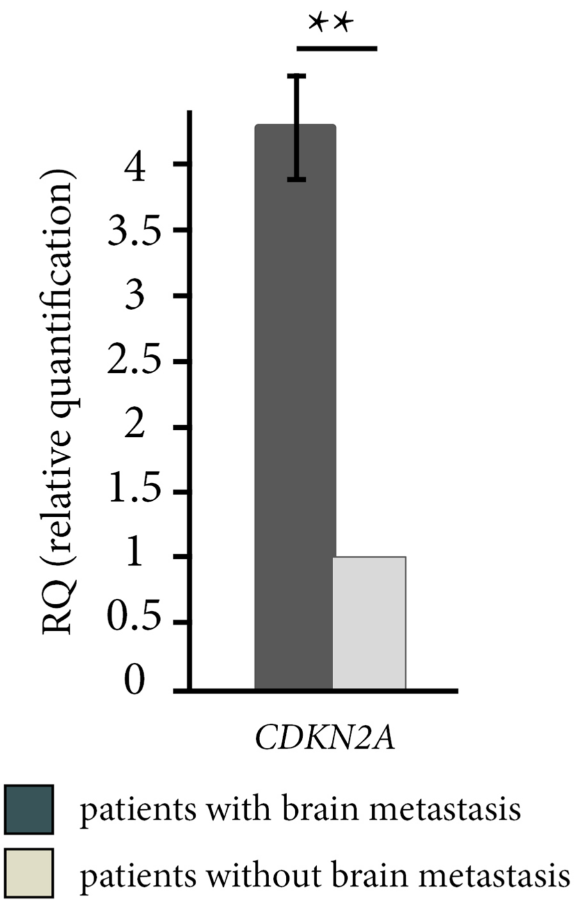

Supplementary Figure 2: CDKN2A mRNA expression in metastatic lymph nodes. CDKN2A mRNA expression is significantly higher in metastatic lymph nodes of women with brain metastases than in women without brain metastases. $^{**}p < 0.01$, $^{*}p < 0.05$.

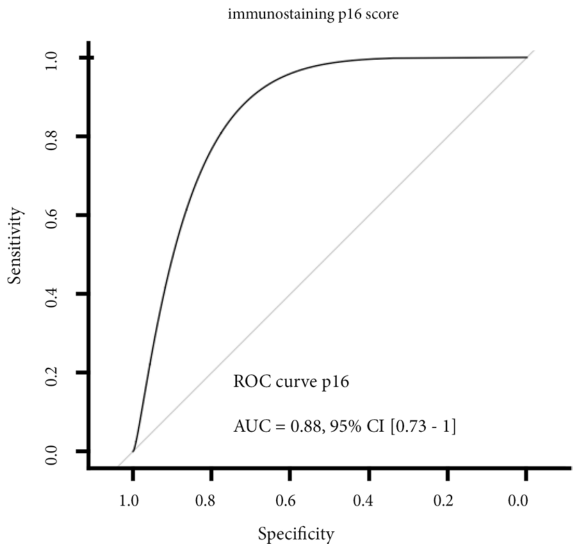

Supplementary Figure 3: ROC curve of the p16 immunostaining score to predict brain metastasis occurring within 3 years following initial diagnosis. Sensitivity and specificity were computed for each value of the quantitative immunohistochemical staining score. ROC: receiver operating characteristics AUC: area under the curve CI: confidence interval.

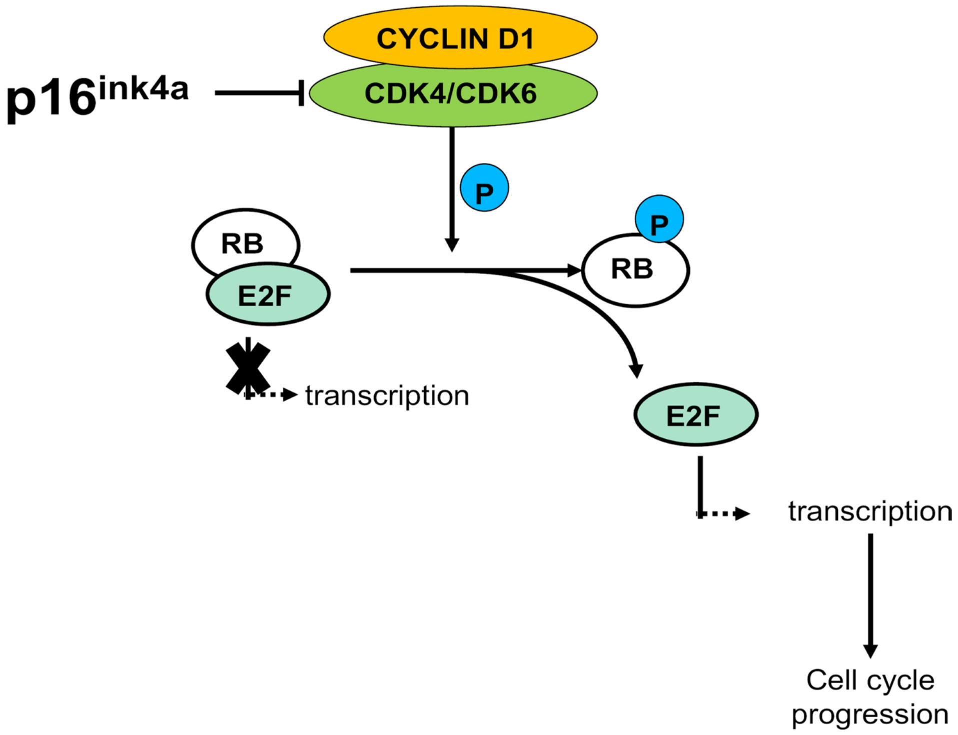

Supplementary Figure 4: RB signaling pathway. p16^{ink4a} inhibits cyclin-dependent kinases CDK4 and CDK6, and prevents phosphorylation of retinoblastoma tumor suppressor (RB). RB represses the activity of the E2F family of transcription factors and blocks cell cycle progression.

Supplementary Table 1: Individual characteristics of the 28 patients with metastatic lymph nodes

N°	Brain metastases	Histological sub-type		Stage at initial diagnosis	Delay between initial diagnosis and metastatic relapse (months)	Metastatic sites at first relapse	Clinical state at the time of analysis
		TNBC	HER2				
1	**Yes**	**Yes**	No	T2N3M0	15	Liver	Dead
2	**Yes**	**Yes**	No	T3N1M0	23	Liver, bone	Dead
3	**Yes**	**Yes**	No	T4dN1M0	6	Brain	Dead
4	**Yes**	**Yes**	No	T2N0M0	17	Liver, lung, lymph node	Dead
5	**Yes**	**Yes**	No	T4dN3M1	0	Lung	Dead
6	**Yes**	**Yes**	No	T2N3M1	0	Bone, brain	Dead
7	**Yes**	**Yes**	No	T3N1M0	17	Brain	Dead
8	**Yes**	**Yes**	No	T2N0M0	14	Bone, lung, lymph node	Dead
9	**Yes**	No	**Yes**	T2N1M0	57	Liver	Dead
10	**Yes**	No	**Yes**	T2N0M0	84	Lung, lymph node	Dead
11	**Yes**	No	**Yes**	T2N2M1	0	Lung	Dead
12	**Yes**	No	**Yes**	T4dN3M1	0	Bone	Dead
13	**Yes**	No	**Yes**	T4dN2M1	0	Bone	Dead
14	No	**Yes**	No	T4bN2M1	0	Liver, bone	Dead
15	No	**Yes**	No	T3N1M0	30	Lung	Dead
16	No	**Yes**	No	T3N1M0	29	Lung	Dead
17	No	**Yes**	No	T2N0M0	67	Lung, lymph node	Dead
18	No	**Yes**	No	T2N0M0	132	Liver, lung, lymph node	Dead
19	No	**Yes**	No	T3N2M0	22	Liver, lung	Dead
20	No	**Yes**	No	T1N0M0	73	Bone, lung, lymph node	Dead
21	No	**Yes**	No	T1N1M0	86	Liver, bone, lung	Dead
22	No	No	**Yes**	T4dN2M1	0	Liver, bone	Alive without disease
23	No	No	**Yes**	T4bN2M1	0	Lung	Alive without disease
24	No	No	**Yes**	T2N3M0	129	Bone	Alive without disease
25	No	No	**Yes**	T4dN1M1	0	Bone	Alive without disease
26	No	No	**Yes**	T3N2M1	0	Liver, bone	Dead
27	No	No	**Yes**	T2N1M0	24	Skin, lung, lymph node	Alive without disease
28	No	No	**Yes**	T2N1M0	76	Skin, liver, lung	Dead

Supplementary Table 2: Characteristics of patients with available published transcriptomic data

	**HG-U133A GeneChip arrays *N* = 583**	**HG-U133A plus 2.0 GeneChip arrays *N* = 204**
HER2 histological sub-type *N* (%)		
Yes	26 (4.5)	54 (26.5)
No	62 (10.6)	150 (73.5)
ND	495 (84.9)	
Triple negative histological sub-type *N* (%)	39 (6.7)	60 (29.4)
Occurrence of brain metastases *N* (%)		
Yes	86 (14.7)	16 (7.8)
No	480 (82.3)	188 (92.2)
ND	17 (3)	

ND: not determined.

Supplementary Table 3: Characteristics of the 24 patients of the validation series

	**Group With brain metastases *N* = 16**	**Group Without brain metastases *N* = 8**
Median age at diagnosis (range)	46 years (33–62)	59 years (32–72)
Median survival from diagnosis of metastatic disease (range)	30.5 months (2.4–141.9)	34.3 months (15.6–79.7)
Histological sub-type N (%)		
HER2-overexpressed	8 (50)	5 (62.5)
Triple negative	8 (50)	3 (37.5)
Site of metastastic biopsy sample *N* (%)		
Lymph node	16 (100)	8 (100)

Supplementary Excel File 1: SamplInfoA_605. See Supplementary_Excel_File_1

Supplementary Excel File 2: SamplInfoP2_204. See Supplementary_Excel_File_2
